# Multicomponent Analysis of Liuwei Dihuang Pills by a Single Marker Quantification Method and Chemometric Discrimination of Fingerprints

**DOI:** 10.1155/2023/6648668

**Published:** 2023-09-15

**Authors:** Lin Yang, Yan Li, Yuanfang Hou, Yongfu Wu, Lihong Tan, Zhenqiang Mu, Zhaojing Zhu, Dan He

**Affiliations:** ^1^Chongqing Pharmaceutical Preparation Engineering Technology Research Center, Chongqing Medical and Pharmaceutical College, Chongqing 401331, China; ^2^Traditional Chinese Medicine Factory Co. Ltd., Taiji Group Chongqing, Chongqing 402284, China; ^3^Chongqing Research Center for Pharmaceutical Engineering, College of Pharmacy, Chongqing Medical University, Chongqing 400016, China

## Abstract

An effective and comprehensive quality evaluation method for Liuwei Dihuang pills (LDP) was established by the simultaneous determination of 8 active components in LDP by the quantitative analysis of multicomponents by single marker (QAMS) method and high-performance liquid chromatography (HPLC) fingerprint combined with chemometrics. These 8 active components were determined by QAMS and the external standard method (ESM), and the quantitative results of the two methods were compared to validate the accuracy and feasibility of the QAMS method. 8 active components showed good linear relationships within their ranges, whose average recoveries were 99.7∼102.3%. No significant difference was found (*P* > 0.05) in the quantitative results determined by QAMS and ESM. Furthermore, the fingerprint of LDP was also established, with 11 common peaks identified, and the similarity of the fingerprints of 21 batches of LDP was greater than 0.95. The 21 batches of LDP were basically divided into 3 groups by hierarchical cluster analysis (HCA) and principal component analysis (PCA), and 3 differential markers were screened out by orthogonal partial least squares discriminant analysis (OPLS-DA). The established QAMS method is accurate, economical, fast, and convenient and can simultaneously determine the content of 8 active components in LDP. HPLC fingerprint combined with chemometric analysis more comprehensively evaluated the quality consistency of different batches of LDP and analyzed the markers that cause quality differences between batches. It can provide a scientific basis and reference of quality consistency evaluation for the manufacturers and drug regulatory departments of the preparation.

## 1. Introduction

Liuwei Dihuang pills (LDP) is a classic prescription for nourishing yin and tonifying kidney. It is composed of 6 herbs: Rehmannia glutinosa, Cornus officinalis (manufactured), Cortex Moutan, Rhizoma Dioscoreae, Poria cocos, and Alisma orientalis [1]. LDP has thousands of years of clinical application history in China. It is used to treat kidney yin deficiency, dizziness, tinnitus, soreness and weakness of waist and knees, bone steaming and hot flashes, essence, and qi night sweats. In addition, it has anti-inflammatory, hypoglycemic, antioxidant, enhancing immune function, delaying aging, anticancer, and other effects [2]. Recently, LDP is more and more extensively used and has played a good role in the prevention, treatment, and adjuvant treatment of tumors [3]. Compared with western medicine, LDP combined with western medicine can better reduce systolic blood pressure and diastolic blood pressure and has advantages in clinical efficacy and antihypertensive effect [4].

The quality evaluation indicators of LDP in the “Chinese Pharmacopoeia” (2020 edition) are 3 active ingredients: morroniside, loganin, and paeonol [1], which are too few to reflect its quality comprehensively and accurately. Some studies have also used the external standard method (ESM) for the simultaneous determination of multiple active ingredients of LDP by high-performance liquid chromatography (HPLC) [5–9], but the ESM requires a large number of standards and is costly. The application of ESM and internal standard methods will be limited when the reference is insufficient or unstable, especially in multicomponent quantitative and quality control [10].

**Figure 1:**
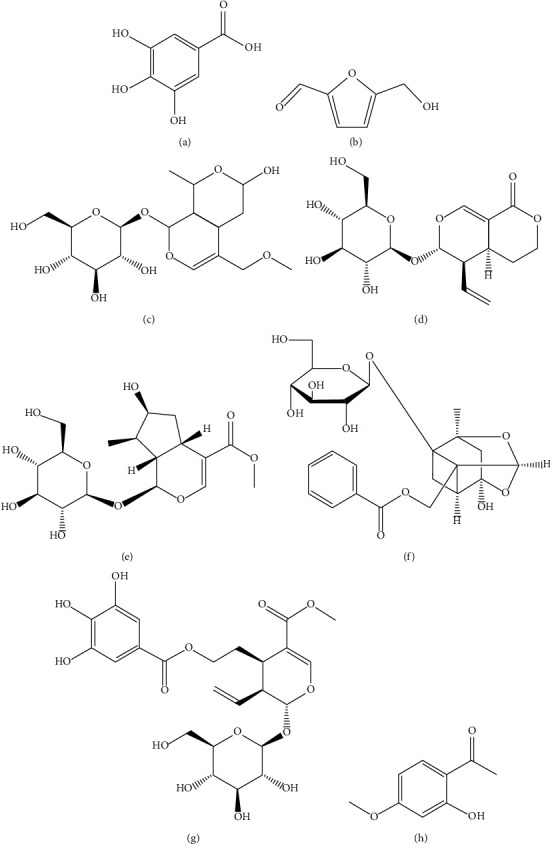
The structures of the 8 quality control markers: (a) gallic acid, (b) 5-hydroxymethylfurfural, (c) morroniside, (d) sweroside, (e) loganin, (f) paeoniflorin, (g) cornuside, and (h) paeonol.

Quantitative analysis of multicomponents by single marker (QAMS) has been widely used in the quality control of traditional Chinese medicine (TCM) [11–15], but few reports have been published for the preparation of LDP with few quantitative indicators [16, 17]. In this study, based on the comprehensive consideration of the effectiveness [18–23], measurability of the components, and easy availability of the reference substance, the simultaneous quantification of gallic acid, 5-hydroxymethylfurfural (5-HMF), morroniside, sweroside, loganin, paeoniflorin, and cornuside in LDP was realized by using HPLC with paeonol as the internal standard through the QAMS method. The structures of the 8 compounds are shown in Figure 1. Among the 8 active components, gallic acid, morroniside, loganin, sweroside, and cornuside were reported to be effective components of Cornus officinalis. It has the effects of regulating immunity, reducing blood sugar, and antiarrhythmic [24–26]. Paeonol and paeoniflorin are considered to be the effective components of Cortex Moutan, which has the functions of reducing blood sugar, antiatherosclerosis, protecting myocardial ischemic tissue, and antitumor [27, 28]. 5-HMF is considered to be an effective ingredient of Rehmannia glutinosa, Fructus Corni, Cortex Moutan, and Rhizoma Dioscoreae. Rehmannia glutinosa has the effects of decreasing blood sugar, antiaging, antitumor, promoting hematopoietic function, and enhancing immunity [29, 30]. Yam has hypoglycemic, hypolipidemic, and antioxidant effects [31, 32].

The TCM fingerprint can reflect the results of a multicomponent and multitarget synergistic treatment of TCM based on the overall understanding of its chemical compositions. The internal quality of the compound can be reflected to a certain extent by identifying the fingerprint, so as to control the overall quality of TCM compound preparations [33, 34]. Chemical pattern recognition is considered to be a more objective and effective method for identifying the specific consistency and stability of TCM products and evaluating the determination of single or multiple markers. Chemometric analysis methods such as similarity analysis, hierarchical clustering analysis (HCA), and principal component analysis (PCA) are widely used in chemical classification and chromatographic profile analysis [35]. Currently, there is no literature report on the quality consistency of LDP and the screening of quality differential markers between various batches by fingerprint combined with chemometrics.

In this research, to establish an efficient, economical, and practical quality evaluation method for LDP, 11 common peaks were identified in the established fingerprint, and chemometrics approaches were used to extract the covered information and knowledge from chemical systems. The method is accurate, reliable, simple, and robust. Combined with QAMS, it can comprehensively and scientifically evaluate the quality of LDP and provide a scientific basis and reference for the quality consistency control of LDP for manufacturers and drug regulatory authorities.

## 2. Experimental

### 2.1. Instruments

Quantitative HPLC analysis was performed on Waters e2695 high-performance liquid chromatograph with Waters 2998 PDA detector, Agilent 1260 Infinity II, Shimadzu Nexera X2 liquid chromatography. The chromatographic columns were Phenomenex Luna C18 column (4.6 mm × 150 mm, 5 *μ*m), Waters Sunfire C18 column (4.6 mm × 150 mm, 5 *μ*m), and Wondasil C18-WR column (4.6 mm × 150 mm, 5 *μ*m). FA2204B 1/10,000 balance was provided by Shanghai Tianmei Balance Instrument Co., Ltd. (Shanghai, China), SB25-12DTN ultrasonic instrument was acquired from Ningbo Scientz Biotechnology Co., Ltd. (Ningbo, China), and Milli-Q integral-3 ultrapure water machine (Merck Millipore, Germany) was also used in quantitative analysis.

### 2.2. Chemical Reagents

Gallic acid (purity 90.8%, 110831-201605), 5-HMF (purity 99.2%, 111626-201912), morroniside (purity 96.3%, 111998-201602), sweroside (purity 100%, 111742-200501), loganin (purity 99.0%, 111640-201808), paeoniflorin (purity 95.1%, 110736-201943), and paeonol (purity 99.9%, 110708-201407) were all purchased from China Institute for Food and Drug Control; cornuside (purity 98.0%, 19080905) was purchased from Chengdu Pufei De Biotech Co., Ltd.

Methanol and acetonitrile were chromatographically pure and purchased from Chengdu Kelong Chemical Co., Ltd.; phosphoric acid was chromatographically pure and purchased from Chongqing Chuandong Chemical (Group) Co., Ltd.; and water was ultrapure. 21 batches of LDP (concentrated pills) were purchased from Taiji Group Chongqing No. 2 Chinese Medicine Factory Co., Ltd. (lot numbers: 2104086, 2105103, 2105109, 2107154, 2107155, 2110224, 2202033, 2203039, 2203040, 2203041, 2203042, 2203043, 2204062, 2205069, 2205071, 2205075, 2205076, 2205078, 2205079, 2205080, and 2206086, named S1–S21).

### 2.3. Software Methods

“Similarity Evaluation System for Chromatographic Fingerprint of Traditional Chinese Medicine” (version 2012A, Chinese Pharmacopoeia Commission, Beijing, China) was used to establish the fingerprint and analyze the similarity by importing the chromatogram of 21 batches of LDP. “Excel” was used to conduct the radar plot analysis. “SPSS 23.0” data analysis software was used to cluster samples. “SIMCA 14.1” software was used to identify the principal component.

### 2.4. Chromatographic Conditions

**Figure 2:**
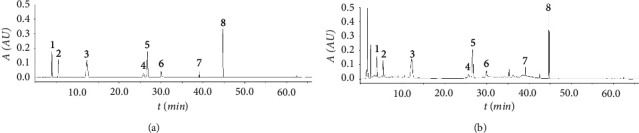
HPLC chromatograms of mixed standards (a) and Liuwei Dihuang pills sample (b). Note. (1) Gallic acid. (2) 5-hydroxymethylfurfural. (3) Morroniside. (4) Sweroside. (5) Loganin. (6) Paeoniflorin. (7) Cornuside. (8) Paeonol.

A Phenomenex Luna C18 column (4.6 mm × 150 mm, 5 *μ*m) was used based on the properties of 8 compounds. The mobile phase was acetonitrile (A)-0.2% phosphoric acid (B) with gradient elution (0∼5 min, 5% A⟶8% A; 5–20 min, 8% A; 20∼35 min, 8% A⟶20% A; 35∼45 min, 20% A⟶60% A; 45∼55 min, 60% A; 55∼56 min, 60% A⟶95% A; 56∼60 min, 95% A; 60∼61 min, 95% A⟶5% A; 6∼66 min, 5% A). The flow rate was 1.0 mL·min^−1^, the detection wavelength was 240 nm, the column temperature was 30°C, and the injection volume was 20 *μ*L. Under the abovementioned chromatographic conditions, the chromatograms of mixed standard solution and sample solution are shown in Figure 2.

### 2.5. Preparation of Mixed Standard Solution and Sample Solution

A series of concentrations of the mixed solutions were prepared by dissolving the accurately weighed and appropriate amounts of each reference in 70% methanol. The mixed solutions contained 17.51∼437.76 *μ*g·mL^−1^ gallic acid, 10.44∼261.12 *μ*g·mL^−1^ 5-HMF, 17.48∼437.04 *μ*g·mL^−1^ morroniside, 3.33∼83.20 *μ*g·mL^−1^ sweroside, 14.90∼372.48 *μ*g·mL^−1^ loganin, 8.24∼205.92 *μ*g·mL^−1^ paeoniflorin, 1.21∼30.22 *μ*g·mL^−1^ cornuside, and 20.59∼514.80 *μ*g·mL^−1^ paeonol.

LDP was crushed by a multifunction pulverizer for 2 min. 1.65 g of fine powder of LDP was accurately weighed and placed in a 50 mL measuring flask, and then about 40 mL of 70% methanol was added. The mixture was sonicated for 1 h, cooled, diluted with 70% methanol to scale, and filtered with a 0.22 *μ*m microporous membrane, and the subsequent filtrate was taken as the sample solution.

### 2.6. Validation of Analytical Method

The mixed standard solution and the sample solution were injected with 20 *μ*L, respectively, and analyzed under the 200–400 nm full scanning wavelength to test the system suitability and specificity. The mixed standard solution was injected with 20 *μ*L to obtain the peak areas, and the linear regression was carried out with the concentration as the abscissa (*X*) and the peak area as the ordinate (*Y*). The mixed standard solution was injected 6 times continuously to evaluate the precision. The same batch of LDP (S3) was taken to prepare 6 sample solutions in parallel and determined to investigate the repeatability of the established method. The stability was tested by injecting the same sample solution (S3), respectively, after being placed at room temperature for 0, 4, 8, 12, 18, 24, and 36 h. The known contents of LDP (S3) powder were precisely weighed and placed in a 50 mL volumetric flask (*n* = 9) and then divided into three groups on average. Each group was precisely added with about 80%, 100%, and 120% of the standard solution equivalent to the content of each component in the sample. The peak area was recorded, and the recovery of each component was calculated.

### 2.7. Establishment of HPLC Fingerprint and Chemometrics Analysis

The 21 batches of LDP were prepared according to the preparation method of the sample solution. The samples were determined, and the chromatograms were recorded. After the unified integration of all chromatograms, the obtained chromatograms were imported into the “Similarity Evaluation System for Chromatographic Fingerprint of Traditional Chinese Medicine (2012 Edition)” software in AIA format to establish the HPLC fingerprint. The similarity of fingerprints of 21 batches of LDP was also calculated by the abovementioned software. Taking the peak area of 11 common peaks of 21 batch samples as variables, using SPSS 23.0 data analysis software, the intergroup connection method and Euclidean distance were selected for systematic HCA. The peak areas of common peaks in 21 batches of samples were imported into SIMCA 14.1 software for PCA, and the data were fitted by Ctr's scaling method to obtain the corresponding score map. In order to find the characteristic components that cause quality differences between various batches, SIMCA. 14.1 was used to conduct orthogonal partial least squares discriminant analysis (OPLS-DA).

## 3. Results and Discussion

### 3.1. Methodology Verification

There were chromatographic peaks at the corresponding positions in the chromatograms of the sample solution and the mixed standard solution, and the spectral characteristics of the chromatographic peaks were consistent with those of the standards. The peak purity of each target component was detected, and the results met the requirements (the purity angle was less than the purity threshold). The separation degree of each peak is better than 1.5, and the number of theoretical plates is not less than 3000. As shown in Table 1, each *r* of the 8 components within their linear range was not less than 0.9991, indicating that the method had a satisfactory linear relationship. The RSDs of the 8 components were no more than 0.33%, 0.95%, and 0.92%, demonstrating the instrument had good precision, the method had satisfactory repeatability, and the sample solution was stable within 36 h at room temperature. The average recoveries of the 8 ingredients were in the range of 99.1%∼102.2% (Table 2), manifesting the presented method was accurate. The abovementioned experimental results indicated that the HPLC method was accurate and reliable for the quantitative analysis of LDP.

#### 3.1.1. Optimization of Chromatographic Condition

The mobile phase composition of this method was mainly referred to the “Chinese Pharmacopoeia” 2020 edition, using an acetonitrile-0.3% phosphoric acid solution for gradient elution. Considering the acidity of the 0.3% phosphoric acid is not conducive to the protection of the chromatographic column, 0.1% and 0.2% phosphoric acid aqueous solutions were also investigated. The results showed that 0.1% phosphoric acid reduced the separation degree by reducing the number of column plates with a peak deformation difference. When 0.2% phosphoric acid was used, the performance of each chromatographic peak was similar to 0.3% phosphoric acid, and the acidity of the mobile phase was weakened, which was beneficial to prolong the life of the column. Consequently, an acetonitrile-0.2% phosphoric acid solution was selected for gradient elution. The chromatographic peaks of each component were scanned at full wavelength using a diode array detector. Gallic acid had the maximum absorption at 216 nm and 271 nm, 5-hydroxymethylfurfural had the maximum absorption at 230 nm and 285 nm, morroniside had the maximum absorption at 241 nm, paeoniflorin had the maximum absorption at 246 nm, loganin had the maximum absorption at 237 nm, paeoniflorin had the maximum absorption at 232 nm and 274 nm, cornuside had the maximum absorption at 218 nm and 273 nm, and paeonol had the maximum absorption at 211 nm, 228 nm, 274 nm, and 312 nm. Due to the different contents of each component in the sample, taking into account the response value of each component, 240 nm was selected as the detection wavelength. Under the chromatographic conditions, the chromatogram showed a smooth baseline and an efficient separation degree of the target components which meet the system suitability requirements.

### 3.2. Establishment of the QAMS Method

#### 3.2.1. Calculation of Relative Correction Factor (RCF)

The dominant advantage of QAMS is that only one standard is needed in the daily inspection work to quantitatively determine the multicomponent as long as the method was established. It greatly saves the number of standards and has the characteristics of simplicity, easy operation, and low cost. The selection of internal standards should be in accordance with the principles of stability, low price, availability, low toxicity, and effectiveness. After comparison, among the components to be measured, paeonol had high content, stable property, and optimal chromatographic peak shape and are nontoxic, and the standard was the cheapest and easy to obtain, and hence, paeonol was selected as the internal standard. The relative correction factors calculated under various instruments and columns with paeonol as the internal standard were satisfactory, manifesting that the established QAMS method had great durability and stability.

The relative correction factors of gallic acid, 5-HMF, morroniside, sweroside, loganin, paeoniflorin, and cornuside were calculated with paeonol as the internal standard. The results are shown in Table 3.

The formula is as follows:

(1) $$\begin{array}{c} f_{s/i}=\frac{f_{s}}{f_{i}}\\ =\frac{\left(A_{s}✕C_{i}\right)}{\left(A_{i}✕C_{s}\right)}\text{,} \end{array}$$

where *A*_*s*_, *C*_*s*_, *A*_*i*_, and *C*_*i*_ represent the peak area of the internal standard, the concentration of the internal standard, the peak area of component *i*, and the concentration of component *i*. According to formula (1), the concentration of a component *i*(*C*_*i*_) can be calculated by using the following formula:

(2) $$\begin{array}{c} C_{i}=f_{s/i}\times C_{s}\times \frac{A_{i}}{A_{s}}\text{.} \end{array}$$

#### 3.2.2. The Influence of Different Instruments and Columns

The mixed standard solution was injected to investigate the effect of Waters e2695, Agilent 1260 Infinity II, Shimadzu Nexera X2 liquid chromatography, Phenomenex Luna C18 column (4.6 mm × 150 mm, 5 *μ*m, No. 1#), Waters Sunfire C18 column (4.6 mm × 150 mm, 5 *μ*m, No. 2#), and Wondasil C18-WR column (4.6 mm × 150 mm, 5 *μ*m, No. 3#) on the relative correction factor. The results are shown in Table 4; the RSD values of all RCFs under different experimental conditions were less than 5%, indicating that the QAMS had good durability and stability. Different instruments and chromatographic columns had no significant influence on the relative correction factors of each component.

#### 3.2.3. The Location of the Analytes' Chromatographic Peaks

With the paeonol chromatographic peak as the reference, the relative retention times (RRT) of gallic acid, 5-hydroxymethylfurfural, morroniside, sweroside, loganin, paeoniflorin, and cornuside were calculated, respectively. The reproducibility of the RRT of each component under different HPLC conditions and different chromatographic columns was investigated. As shown in Table 5, the RRT fluctuation of each component was small. In this study, the relative retention time was used as the positioning index of the target chromatographic peak of QAMS, and the position of the target peak was further accurately determined by combining the UV absorption characteristics of each chromatographic peak in the sample solution.

### 3.3. Sample Determination

The concentrations of the 8 effective components in 21 batches of LDP determined by the ESM and QAMS methods are listed in Table 6. The content of 8 active components in 21 batches of LDP was different. Paeonol had the highest content, followed by 5-HMF, morroniside, and loganin. Paeoniflorin, sweroside, and cornuside had relatively low content, and paeoniflorin was not detected in some batches. Among the 21 batches of samples, gallic acid had the largest content difference, followed by paeoniflorin, cornuside, 5-HMF, loganin, and paeonol. It shows that there are some differences in the content of each active component between different batches, which may be related to the source of medicinal materials and the extraction and production processes of medicinal materials. For manufacturers, the source of herbs should be strictly controlled, and the extraction and production processes should be standardized to ensure the consistency of drug quality.

### 3.4. Evaluation of the QAMS and ESM Methods

The 21 batches of LDP were prepared as a sample solution and injected for determination. The contents of 8 components were calculated by ESM and QAMS. The difference between the two methods was analyzed by the *t* test. As shown in Table 6, there is no significant difference between the content values measured by the ESM and QAMS methods. The results of ESM and QAMS were tested by SPSS 17 statistical software. The results are shown in Table 6. The confidence interval was 95%, and the *P* values were all greater than 0.05, indicating that there was no significant difference in the content measured by the two methods, indicating that QAMS can be well used for the simultaneous determination of eight active components. The QAMS is a reliable and convenient method for the determination of multicomponent content, especially in the absence of reference substances. This strategy reduced the experimental cost and assay time, and the QAMS method was applied accurately to the quantitative analysis of LDP.

### 3.5. Establishment and Similarity Evaluation of HPLC Fingerprint

**Figure 3:**
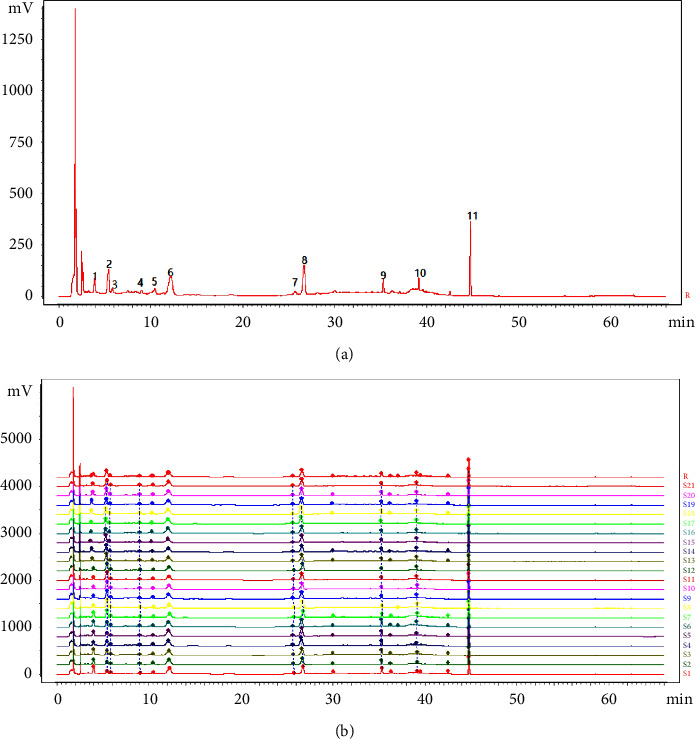
HPLC fingerprint of 21 batches of LDP: (a) identification of chemical common peaks (b) HPLC fingerprints and reference fingerprint (*R*).

The chromatogram of the S1 sample with a steady baseline, good peak shape, and separation was selected as the reference. The multipoint correction method was used to match the whole peak of the chromatogram with the time window width of 0.2, the control fingerprint (*R*) was generated by the median method, the HPLC fingerprint of 21 batches of samples was generated, and 11 common peaks were identified (Figure 3). Among the 11 common peaks, peaks 1, 2, 6, 7, 8, 10, and 11 were identified as gallic acid, 5-hydroxymethylfurfural, morroniside, sweroside, loganin, cornuside, and paeonol.

Using the chromatogram of the S1 sample as the reference chromatogram, the RSDs of the retention time of each common peak were less than 3.19%, and the RSDs of each common peak area were distinct. The similarity results of 21 batches of samples were more than 0.95, indicating that the consistency of LDP quality was good and the chemical composition of LDP was similar, but the content of common peak components varied greatly among batches. Consequently, chemical pattern recognition should be carried out to reflect the intrinsic quality of LDP more objectively.

### 3.6. Hierarchical Cluster Analysis

**Figure 4:**
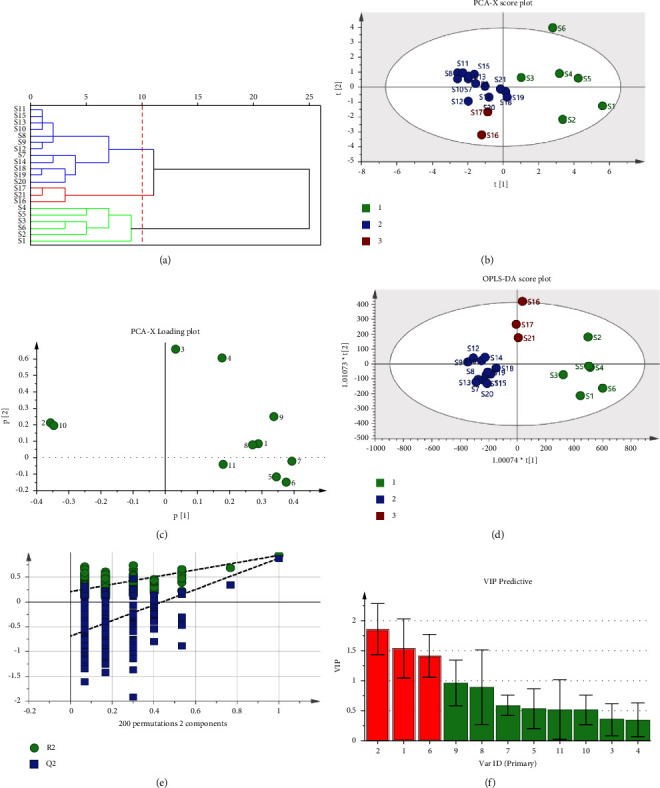
(a) Hierarchical cluster analysis dendrogram. (b) Score plot of PCA. (c) Loading plot of PCA. (d) Score plot of OPLS-DA. (e) Permutation test of OPLS-DA. (f) VIP values of common components.

HCA, which is to say, “objects are clustered by class,” is a practical method in multivariate statistics [36]. It mainly achieves the purpose of classification by using the principle that the same kind of samples are similar to each other, and the distance of similar samples in multidimensional space is smaller, while the distance of different samples in multidimensional space is larger. HCA was performed on SPSS 23.0 data analysis software by selecting the intergroup connection method and Euclidean distance with the peak area of 11 common peaks in 21 batches of LDP as variables. As shown in the Figure 4(a), the Euclidean distance “10” was selected as the judgment basis, 21 batches of samples were clustered into 3 categories, S1–S6 were clustered into one category, S16, S21, and S17 were clustered into the same category, and the rest of the batches were clustered into another category. Adjacent batches of LDP are relatively close in production time, and the harvesting season, sources of medicinal materials, and processing methods of the prescription medicinal materials may be more similar, so they are more likely to be grouped together. Since it is impossible to guarantee the production conditions are exactly the same in every production, it is reasonable to have some quality differences between samples from batch to batch. In addition, differences in the quality of the samples may be influenced by the source of the herbs, the production process, and the conditions of transport and storage. So the production of Chinese patent medicines should be considered comprehensively, taking into account various variable factors, so that the production quality of each batch of medicinal materials is consistent.

### 3.7. Principal Component Analysis

PCA is an unsupervised pattern recognition method. On the basis of the dimension reduction idea, it can transform the multiple indicators into several independent synthetic indicators which contain most of the information in the original ones. After the transformation, the data matrix is simplified, the dimension is reduced, and a few principal components linearly combined by the original variables are found, which is convenient for the multivariate statistical method of extracting chemical information [37]. The results of the scatter plot of principal component scores (Figure 4(b)) showed that the contribution rates of principal component 1 and principal component 2 are 53.9% and 19.3%, respectively, and the cumulative contribution rate reaches 73.2%. Therefore, the two principal components can comprehensively reflect the total compositional content of the peak. The 21 batches of samples can be divided into three groups: samples S16 and S17 were in one group, S1–S6 belonged to another group, and the rest of the samples were in another group. Except for S21, the results of PCA are generally consistent with the results of HCA. The results indicate that samples produced at similar times have high similarity. The scatter diagram of the principal component loading (Figure 4(c)) revealed the proportion of each chromatographic peak in the principal component, where the abscissa represents the loading of each substance on the principal component 1 and the ordinate represents the loading of each substance on the principal component 2. The farther the peak is from the *Y*-axis, the greater the contribution to the principal component 1, and peak 2 (5-HMF), peak 6 (morroniside), peak 7 (sweroside), and peak 10 (cornuside) had an important contribution to the principal component 1 when the absolute value of the *X*-axis distance was limited to 3.5. The farther the peak is from the *X*-axis, the greater the contribution to principal component 2, and peaks 3 and 4 had an important contribution to the principal component 2 when the absolute value of the *Y*-axis was bounded by 3.5. 5-HMF, morroniside, sweroside, and cornuside were all bioactive components in LDP and evaluated in QAMS.

### 3.8. Orthogonal Partial Least Squares Discriminant Analysis

OPLS-DA is a supervised pattern recognition method for multivariate statistics of massive data to explore the differences of indicators between groups [38]. As shown in Figures 4(d)–4(f), the fitting parameters of the established model were *R*^2^*X* = 0.925, *R*^2^*Y* = 0.820, and the prediction parameter *Q*^2^ = 0.671, which means that the model predicts the variables well. As exhibited in Figure 4(d), except for the S16, the 21 batches of LDP were all within the 95% confidence interval. S1–S6 were clustered into one category, S16, S17, and S21 were clustered into one category, and the rest of the samples were clustered into one category, which is basically consistent with the HCA. The results of the 200 permutations shown in Figure 4(f) demonstrated that the *Y*-axis intercept of the *R*^2^ fitting line was 0.211 and less than 0.3, indicating that the established model is reliable. The *Y*-axis intercept of the *Q*^2^ fitting line was −0.688 and *Q*^2^ was a negative number. The *Q*^2^ and *R*^2^ values on the right were higher than those on the left, manifesting that the established model does not have overfitting and has good predictive ability. It can be seen from Figure 4(f) that the differential components in LDP were screened according to the variable influence on projection (VIP) results, among which peak 1 (gallic acid), peak 2 (5-HMF), and peak 6 (VIP value of monoglucoside) were greater than 1, indicating that gallic acid, 5-HMF, and morroniside were the main quality markers affecting the quality consistency of LDP. Gallic acid and morroniside were reported to be effective components of Cornus officinalis, and 5-HMF was considered to be an effective ingredient of Rehmannia glutinosa. It was suggested that manufacturers should control the quality consistency of Cornus officinalis and Radix Rehmanniae to ensure the consistency of LDP quality. It is interesting that in the prescription of LDP, Rehmannia glutinosa is the principal drug and Cornus officinalis is the adjuvant drug. The research results just showed that the quality control of the principal drug and adjuvant drug should be more important.

### 3.9. Radar Plot Analysis

**Figure 5:**
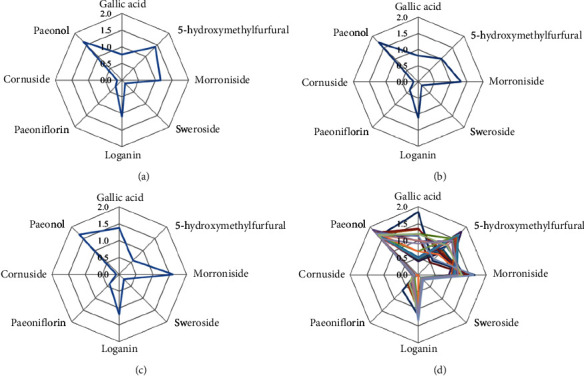
Radar plots showing the difference contents in terms of 8 components in different batches LDP samples: (a) G1; (b) G2; (c) G3; and (d) the distribution of the chemical composition patterns of 21 batches of LDP.

Radar plot is an important descriptive tool for multivariate data. Generally speaking, radar plots are a circular graphic approach that projects a series of spokes or rays from the center point, each ray represents different variable labels [39]. The determination results of 21 batches of LDP were analyzed by radar plot. Figures 5(a)–5(c) show the means of the 8 components in G1–G3 in the HCA, respectively. The content changes of components from G1 to G3 were different. Gallic acid, morroniside, sweroside, loganin, and paeoniflorin are gradually increasing, among which the contents of gallic acid, sweroside, and paeoniflorin were increasing significantly, and the contents of 5-HMF and cornuside were gradually decreasing. The content of paeonol had little difference among the three groups, and there was no obvious change trend. The results of OPLS-DA showed that gallic acid, 5-HMF, and morroniside were the main markers affecting the quality of LDP. From Figure 5(d), it can also be found that gallic acid, 5-HMF, and morroniside were the 3 components with the greatest variation in content among the 8 active components. It was suggested that the components with large content differences may be the main quality markers that affect the quality differences of different batches of LPD.

## 4. Conclusions

The established QAMS method used paeonol as the internal standard, and the RCF calculation method was used to determine the content of 8 active components in 21 batches of LDP, and the methodology was verified. The RCF values of different chromatographic columns and instruments were stable. The results showed that there was no significant difference between QAMS and ESM. The QAMS method is economical and fast and can evaluate the quality of LDP more comprehensively and scientifically than the quality control index of only 3 components in the existing standard.

The analytical method proposed in this paper was established for the quality control of LDP for the first time. The HPLC fingerprint of LDP was also established with 11 common peaks were identified and the similarities of fingerprints were greater than 0.95, demonstrating the quality of LDP is consistent between different batches. The 21 batches of LDP were basically divided into 3 groups by HCA and PCA, and OPLS-DA screened out 3 differential markers in various batches. HPLC fingerprint combined with chemometrics information more comprehensively evaluated the quality consistency of different batches of LDP and analyzed the markers that caused quality differences between batches. Consequently, it can provide a scientific basis and reference for the quality, consistency, and evaluation of LDP for the manufacturers and drug regulatory authorities of the preparation.

## Figures and Tables

**Table 1:** Methodological validation results.

Component	Regression equation	Linear range (*μ*g·mL^−1^)	*r*	Precision (%)	Repeatability (%)	Stability (%)
Gallic acid	*Y* = 5340*X*+77046	17.51∼437.76	0.9991	0.33	0.93	0.44
5-Hydroxymethylfurfural	*Y* = 8713*X*−27346	10.44∼261.12	0.9993	0.30	0.65	0.59
Morroniside	*Y* = 15870*X*−23082	17.48∼437.04	0.9999	0.24	0.62	0.34
Sweroside	*Y* = 13777*X*−7005	3.33∼83.20	0.9998	0.25	0.61	0.31
Loganin	*Y* = 15671*X*−46175	14.90∼372.48	0.9996	0.30	0.95	0.22
Paeoniflorin	*Y* = 9006*X*−55919	8.24∼205.92	0.9995	0.32	0.78	0.92
Cornuside	*Y* = 13390*X*−4018	1.21∼30.22	0.9995	0.28	0.55	0.49
Paeonol	*Y* = 10030*X*−110.6	20.59∼514.80	0.9998	0.26	0.25	0.13

**Table 2:** Results of recovery test.

Component	Content (mg)	Added (mg)	Determined (mg)	Recovery (%)	Average recovery (%) (*n* = 9)	RSD (%) (*n* = 9)
Gallic acid	2.376	1.847	4.267	102.4	102.2	0.51
	2.371	1.847	4.246	101.5		
	2.370	1.847	4.245	101.5		
	2.371	2.309	4.731	102.3		
	2.369	2.309	4.721	101.9		
	2.376	2.309	4.738	102.3		
	2.381	2.771	5.238	103.1		
	2.365	2.771	5.209	102.7		
	2.372	2.771	5.207	102.3		

5-HMF^*∗*^	1.752	1.425	3.228	103.6	100.9	1.3
	1.748	1.425	3.203	102.1		
	1.747	1.425	3.192	101.4		
	1.748	1.781	3.543	100.8		
	1.747	1.781	3.525	99.9		
	1.752	1.781	3.522	99.4		
	1.756	2.137	3.895	100.1		
	1.744	2.137	3.890	100.4		
	1.749	2.137	3.894	100.4		

Morroniside	2.185	1.801	4.041	103.1	101.6	0.91
	2.180	1.801	4.017	102.0		
	2.179	1.801	4.016	102.0		
	2.180	2.251	4.475	101.9		
	2.178	2.251	4.443	100.6		
	2.185	2.251	4.433	99.8		
	2.190	2.701	4.927	101.3		
	2.175	2.701	4.929	102.0		
	2.182	2.701	4.926	101.6		

Sweroside	0.240	0.166	0.403	97.9	99.1	1.2
	0.239	0.166	0.403	98.3		
	0.239	0.166	0.404	98.8		
	0.239	0.208	0.445	98.9		
	0.239	0.208	0.443	98.2		
	0.240	0.208	0.444	98.3		
	0.240	0.250	0.487	98.9		
	0.239	0.250	0.491	101.2		
	0.239	0.250	0.491	100.9		

Loganin	1.784	1.562	3.351	100.3	101.9	1.5
	1.780	1.562	3.347	100.4		
	1.779	1.562	3.344	100.2		
	1.780	1.952	3.753	101.1		
	1.778	1.952	3.772	102.1		
	1.784	1.952	3.780	102.2		
	1.788	2.343	4.210	103.4		
	1.776	2.343	4.211	104.0		
	1.781	2.343	4.207	103.6		

Paeoniflorin	0.736	0.549	1.280	99.1	100.8	1.7
	0.734	0.549	1.274	98.3		
	0.734	0.549	1.283	100.0		
	0.734	0.686	1.423	100.4		
	0.734	0.686	1.424	100.6		
	0.736	0.686	1.423	100.1		
	0.738	0.824	1.583	102.7		
	0.733	0.824	1.584	103.4		
	0.735	0.824	1.577	102.2		

Cornuside	0.197	0.121	0.319	100.6	100.0	0.67
	0.197	0.121	0.318	100.0		
	0.197	0.121	0.317	100.0		
	0.197	0.151	0.349	100.8		
	0.196	0.151	0.348	100.1		
	0.197	0.151	0.346	98.4		
	0.197	0.181	0.379	100.0		
	0.196	0.181	0.378	100.4		
	0.197	0.181	0.378	99.9		

Paeonol	2.952	2.154	5.141	101.6	102.1	0.82
	2.946	2.154	5.155	102.6		
	2.945	2.154	5.175	103.5		
	2.946	2.692	5.703	102.4		
	2.944	2.692	5.718	103.1		
	2.953	2.692	5.678	101.2		
	2.959	3.230	6.228	101.2		
	2.939	3.230	6.231	101.9		
	2.948	3.230	6.226	101.5		

^*∗*^5-HMF: 5-hydroxymethylfurfural.

**Table 3:** Relative correction factors of various components.

Component	Relative correction factor (no.)							Mean	RSD (%)
	1	2	3	4	5	6	7		
Gallic acid	1.9452	1.9328	1.9331	1.9395	1.9298	1.9276	1.9174	1.9322	0.46
5-Hydroxymethylfurfural	1.2121	1.2127	1.2284	1.2392	1.2380	1.2178	1.2173	1.2236	0.94
Morroniside	0.7162	0.6893	0.7017	0.7019	0.7022	0.7092	0.6931	0.7019	1.3
Sweroside	0.8391	0.8525	0.8448	0.8390	0.8481	0.8635	0.8427	0.8471	1.0
Loganin	0.6805	0.7004	0.7033	0.6807	0.6747	0.6813	0.6848	0.6865	1.6
Paeoniflorin	1.8014	1.7394	1.7492	1.7305	1.7384	1.8010	1.7278	1.7554	1.8
Cornuside	0.8029	0.8038	0.7942	0.7778	0.7913	0.7964	0.7996	0.7951	1.1

**Table 4:** Relative correction factors determined by different instruments and columns.

Instrument	Column	Relative correction factor						
		*f*_*s*1_^*∗*^	*f*_*s*2_^*∗*^	*f*_*s*3_^*∗*^	*f*_*s*4_^*∗*^	*f*_*s*5_^*∗*^	*f*_*s*6_^*∗*^	*f*_*s*7_^*∗*^
Waters e2695	1#	1.9522	1.2036	0.7019	0.8471	0.6865	1.7554	0.7851
	2#	1.9338	1.2376	0.6894	0.8009	0.6871	1.8115	0.7960
	3#	1.8909	1.2409	0.7092	0.8341	0.7009	1.7165	0.8067

Agilent 1260 InfinityII	1#	1.9230	1.2091	0.7032	0.8245	0.6972	1.7976	0.7986
	2#	1.8888	1.2318	0.6873	0.8045	0.6922	1.7268	0.7755
	3#	1.9189	1.2330	0.7006	0.8323	0.6875	1.8120	0.8116

Shimadzu Nexera X2	1#	1.9463	1.2257	0.7285	0.8022	0.6929	1.7630	0.7751
	2#	1.9070	1.2053	0.7092	0.8365	0.6775	1.7702	0.8058
	3#	1.9634	1.2323	0.6920	0.8159	0.6894	1.7195	0.7966

Mean		1.9249	1.2244	0.7024	0.8220	0.6901	1.7636	0.7946
RSD (%)		1.4	1.2	1.8	2.1	0.98	2.1	1.7

^*∗*^ *f*_*s*1_, *f*_*s*2_, *f*_*s*3_, *f*_*s*4_, *f*_*s*5_, *f*_*s*6_, and *f*_*s*7_ are the relative correction factors of gallic acid, 5-hydroxymethylfurfural, morroniside, sweroside, loganin, paeoniflorin, and cornuside, respectively.

**Table 5:** Relative retention times determined by different instruments and columns.

Instrument	Column	Relative retention time						
		RRT1^*∗*^	RRT2^*∗*^	RRT3^*∗*^	RRT4^*∗*^	RRT5^*∗*^	RRT6^*∗*^	RRT7^*∗*^
Waters e2695	1#	0.0860	0.1209	0.2722	0.5762	0.5964	0.6704	0.8736
	2#	0.0947	0.1237	0.2826	0.5863	0.6065	0.6796	0.8752
	3#	0.0954	0.1235	0.2851	0.5635	0.5978	0.6761	0.8742

Agilent 1260 InfinityII	1#	0.0873	0.1220	0.2739	0.5797	0.5993	0.6749	0.8759
	2#	0.0956	0.1232	0.2848	0.5908	0.6090	0.6864	0.8777
	3#	0.0957	0.1221	0.2847	0.5637	0.5956	0.6766	0.8761

Shimadzu Nexera X2	1#	0.0863	0.1178	0.2653	0.5710	0.5919	0.6674	0.8728
	2#	0.0953	0.1208	0.2763	0.5823	0.6032	0.6780	0.8857
	3#	0.0960	0.1211	0.2798	0.5614	0.5966	0.6768	0.8788

Mean		0.0925	0.1217	0.2783	0.5750	0.5996	0.6763	0.8766
RSD (%)		4.8	1.5	2.5	1.9	0.92	0.79	0.44

^*∗*^RRT1, RRT2, RRT3, RRT4, RRT5, RRT6, and RRT7 are the relative retention times of gallic acid, 5-hydroxymethylfurfural, morroniside, sweroside, loganin, paeoniflorin, and cornuside, respectively.

**Table 6:** Contents of 8 components in LDP by ESM^*∗*^ and QAMS^*∗*^.

Lot no.	Gallic acid		5-HMF^*∗*^		Morroniside		Sweroside		Loganin		Paeoniflorin		Cornuside		Paeonol
	ESM	QAMS	ESM	QAMS	ESM	QAMS	ESM	QAMS	ESM	QAMS	ESM	QAMS	ESM	QAMS	ESM
2104086	1.846	1.865	0.588	0.592	1.657	1.644	0.203	0.203	1.183	1.180	0.647	0.644	0.090	0.090	1.619
2105103	1.149	1.161	0.505	0.510	1.489	1.478	0.202	0.202	1.031	1.028	0.301	0.300	0.076	0.076	1.645
2105109	1.339	1.353	0.990	0.998	1.254	1.245	0.136	0.137	1.018	1.016	0.421	0.419	0.112	0.112	1.681
2107154	0.689	0.696	0.851	0.858	1.304	1.295	0.190	0.190	1.246	1.243	—	—	0.093	0.093	1.746
2107155	1.139	1.151	0.651	0.657	1.623	1.611	0.206	0.206	1.340	1.337	0.257	0.256	0.096	0.096	1.736
2110224	1.358	1.372	0.929	0.937	1.411	1.400	0.150	0.150	1.189	1.186	0.303	0.301	0.162	0.162	1.757
2202033	1.205	1.218	1.495	1.507	1.047	1.039	0.104	0.104	0.936	0.934	0.357	0.356	0.148	0.148	1.610
2203039	0.496	0.502	1.791	1.805	0.903	0.896	0.130	0.131	1.007	1.005	/^*∗*^	/	0.160	0.160	1.601
2203040	0.522	0.528	1.666	1.679	1.128	1.120	0.143	0.143	1.120	1.117	/	/	0.172	0.171	1.673
2203041	0.477	0.482	1.585	1.598	0.978	0.972	0.124	0.124	0.991	0.989	/	/	0.152	0.151	1.560
2203042	0.503	0.508	1.659	1.672	1.023	1.015	0.136	0.136	1.057	1.054	/	/	0.162	0.162	1.514
2203043	0.466	0.471	1.516	1.528	1.145	1.137	0.144	0.144	1.132	1.129	/	/	0.179	0.179	1.523
2204062	0.699	0.706	1.656	1.670	1.068	1.060	0.137	0.137	1.076	1.073	0.132	0.131	0.165	0.165	1.518
2205069	0.990	1.000	1.392	1.403	1.135	1.127	0.113	0.113	0.996	0.992	0.306	0.305	0.155	0.155	1.873
2205071	0.478	0.483	1.661	1.675	1.073	1.065	0.124	0.124	1.075	1.072	/	/	0.166	0.168	1.626
2205075	0.397	0.401	0.911	0.919	1.298	1.288	0.146	0.146	1.067	1.064	/	/	0.144	0.143	1.748
2205076	0.454	0.459	1.049	1.057	1.280	1.270	0.148	0.148	1.095	1.092	/	/	0.155	0.154	1.673
2205078	1.004	1.015	1.237	1.248	1.283	1.274	0.138	0.138	1.115	1.113	0.266	0.265	0.143	0.143	1.654
2205079	1.203	1.216	1.207	1.217	1.280	1.270	0.133	0.133	1.085	1.082	0.362	0.360	0.138	0.138	1.647
2205080	0.916	0.925	1.419	1.430	1.173	1.164	0.147	0.147	1.132	1.129	0.190	0.189	0.146	0.146	1.486
2206086	0.508	0.514	1.191	1.201	1.337	1.327	0.162	0.162	1.194	1.191	/	/	0.151	0.151	1.724

Mean	0.849	0.858	1.236	1.246	1.233	1.224	0.148	0.148	1.099	1.096	0.322	0.321	0.141	0.141	1.648
*t*	−0.071		−0.083		0.149		−0.011		0.096		0.025		0.010		
*P*	0.943		0.934		0.882		0.992		0.924		0.980		0.992		

^*∗*^ESM: external standard method, QAMS: quantitative analysis of multicomponents by single marker, and 5-HMF: 5-hydroxymethylfurfural. ^*∗*^“/”: not detected.

## Data Availability

The data used to support the findings of this study are included within the article.

## Abbreviations

LDP: Liuwei Dihuang pills
QAMS: Quantitative analysis of multicomponents by single marker
HPLC: High-performance liquid chromatography
ESM: External standard method
HCA: Hierarchical cluster analysis
PCA: Principal component analysis
OPLS-DA: Orthogonal partial least squares discriminant analysis
TCM: Traditional Chinese Medicine
5-HMF: 5-Hydroxymethylfurfural
RCF: Relative correction factor.
